# Mechanistic Modeling of Maternal Lymphoid and Fetal Plasma Antiretroviral Exposure During the Third Trimester

**DOI:** 10.3389/fped.2021.734122

**Published:** 2021-09-20

**Authors:** Babajide Shenkoya, Shakir Atoyebi, Ibrahim Eniayewu, Abdulafeez Akinloye, Adeniyi Olagunju

**Affiliations:** ^1^Department of Pharmaceutical Chemistry, Obafemi Awolowo University, Ile-Ife, Nigeria; ^2^Department of Pharmacology and Therapeutics, Institute of Systems, Molecular and Integrative Biology, University of Liverpool, Liverpool, United Kingdom; ^3^Department of Pharmaceutical and Medicinal Chemistry, University of Ilorin, Ilorin, Nigeria

**Keywords:** pregnancy, antiretroviral, lymph, pharmacokinetics, PBPK, fetus, adherence

## Abstract

Pregnancy-induced changes in plasma pharmacokinetics of many antiretrovirals (ARV) are well-established. Current knowledge about the extent of ARV exposure in lymphoid tissues of pregnant women and within the fetal compartment is limited due to their inaccessibility. Subtherapeutic ARV concentrations in HIV reservoirs like lymphoid tissues during pregnancy may constitute a barrier to adequate virological suppression and increase the risk of mother-to-child transmission (MTCT). The present study describes the pharmacokinetics of three ARVs (efavirenz, dolutegravir, and rilpivirine) in lymphoid tissues and fetal plasma during pregnancy using materno-fetal physiologically-based pharmacokinetic models (m-f-PBPK). Lymphatic and fetal compartments were integrated into our previously validated adult PBPK model. Physiological and drug disposition processes were described using ordinary differential equations. For each drug, virtual pregnant women (*n* = 50 per simulation) received the standard dose during the third trimester. Essential pharmacokinetic parameters, including Cmax, Cmin, and AUC (0–24), were computed from the concentration-time data at steady state for lymph and fetal plasma. Models were qualified by comparison of predictions with published clinical data, the acceptance threshold being an absolute average fold-error (AAFE) within 2.0. AAFE for all model predictions was within 1.08–1.99 for all three drugs. Maternal lymph concentration 24 h after dose exceeded the reported minimum effective concentration (MEC) for efavirenz (11,514 vs. 800 ng/ml) and rilpivirine (118.8 vs. 50 ng/ml), but was substantially lower for dolutegravir (16.96 vs. 300 ng/ml). In addition, predicted maternal lymph-to-plasma AUC ratios vary considerably (6.431—efavirenz, 0.016—dolutegravir, 1.717—rilpivirine). Furthermore, fetal plasma-to-maternal plasma AUC ratios were 0.59 for efavirenz, 0.78 for dolutegravir, and 0.57 for rilpivirine. Compared with rilpivirine (0 h), longer dose forgiveness was observed for dolutegravir in fetal plasma (42 h), and for efavirenz in maternal lymph (12 h). The predicted low lymphoid tissue penetration of dolutegravir appears to be significantly offset by its extended dose forgiveness and adequate fetal compartment exposure. Hence, it is unlikely to be a predictor of maternal virological failure or MTCT risks. Predictions from our m-f-PBPK models align with recommendations of no dose adjustment despite moderate changes in exposure during pregnancy for these drugs. This is an important new application of PBPK modeling to evaluate the adequacy of drug exposure in otherwise inaccessible compartments.

## Introduction

Pregnancy-induced physiological changes reduce plasma concentrations of antiretrovirals (ARV), especially in the third trimester (1–4). Mother-to-child transmission (MTCT) of HIV is reduced significantly at the standard dose of current ARVs in use (1, 4–7). Cases of perinatal transmission, although not common, and vaginal shedding of HIV RNA among pregnant women with undetectable or low plasma HIV RNA suggest that declining MTCT may not be attributed to low plasma HIV RNA viral load alone (8–10).

The use of ARVs suppresses plasma HIV RNA levels below the limit of detection (11). However, rapid viral rebound in non-adhering patients suggests that replication-competent viruses persist in HIV reservoirs during treatment (12, 13). Suboptimal adherence may therefore cause a viral rebound in pregnant women, which can increase the risk of mother-to-child transmission (MTCT) (14–16). The lymphoid tissues constitute the largest HIV reservoir sites because they are the primary sites for viral replication, and therefore contain a high proportion of viral genetic components and free virions (15, 17, 18). Furthermore, persistent isolates of HIV particles in lymph nodes of patients on active ART also suggest that the virus may be capable of evading lethal ARV concentrations in maternal plasma. This has constituted a major barrier in HIV eradication (12, 19–22). Penetration of ARVs into the lymphatic tissues is crucial for prevention of viral replication, rebound, drug resistance and MTCT (23).

Quantification of drug distribution into the lymphatic system of living persons has not been studied due to the challenges with sample collection. Macaque mass spectrometry imaging, human lymph node mononuclear cells, and human primary lymphoid endothelial cells are methods that have been reported so far in the literature for drug quantification in lymphoid tissues (24–26). Ethical considerations around sample collection and safety concerns limit fetal pharmacokinetics studies before delivery (27). These gaps may be filled through physiologically-based pharmacokinetic (PBPK) modeling and simulation.

Materno-fetal PBPK (m-f-PBPK) modeling strategy has advanced from simple models to using highly representative models that include gestational-age dependent changes in maternal and fetal anatomy and physiology (27–30). M-f-PBPK models have been used to reliably estimate fetal concentrations of emtricitabine, tenofovir, nevirapine, darunavir, efavirenz, and thalidomide (28–30). These predictions sometimes rely on a number of assumptions based on data derived from *in vitro* or animal models in the absence of relevant clinical pregnancy data. However, a robust mechanistic workflow on PBPK models starting from simple non-pregnant models validated with available clinical data to more complex materno-fetal models, often builds confidence in the data output from such models. Applications of such models to HIV tissue reservoirs could support the development of molecules with optimal characteristics for enhanced distribution in HIV eradication studies. There is currently no published description of ARV distribution into lymphoid tissues during pregnancy.

In the current work, we describe the extension of our previous m-f PBPK model (28) to describe the penetration of efavirenz, dolutegravir and rilpivirine into the lymph and fetal plasma during pregnancy.

## Method

### Model Structure and Parameterisation

**Figure 1:**
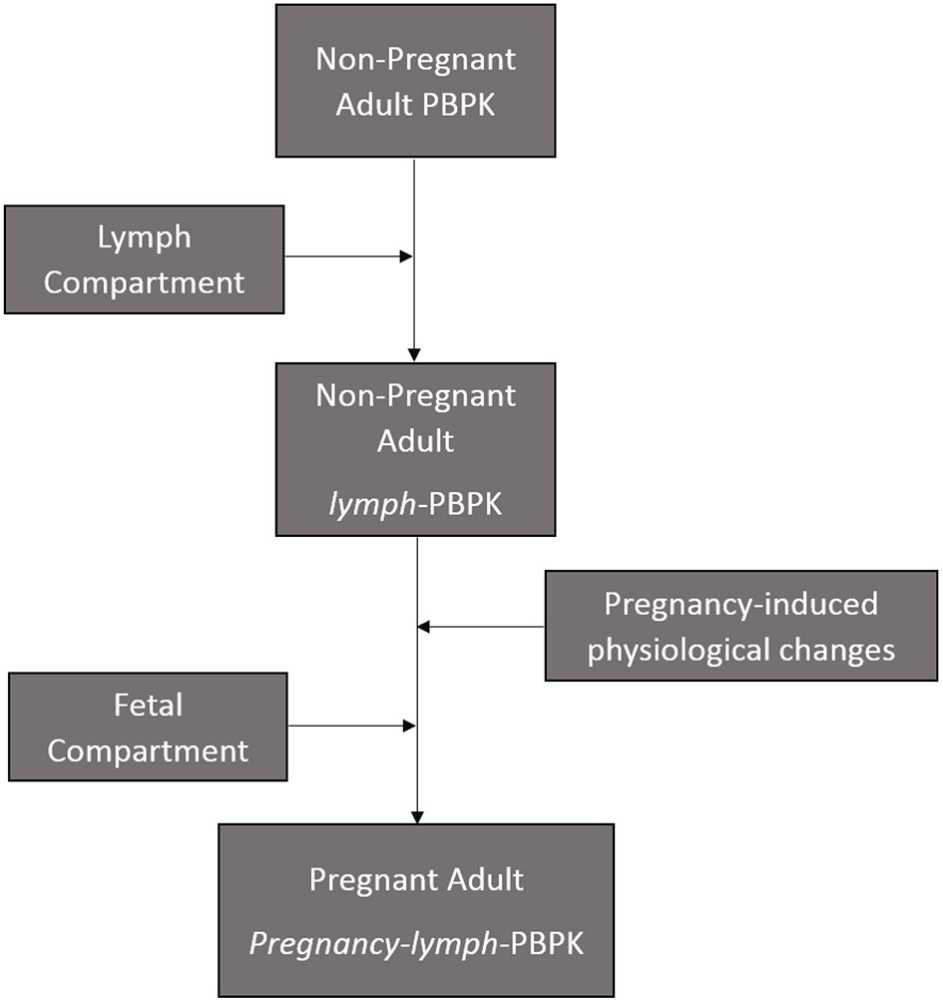
PBPK Model development workflow adopted.

The present model is an extension of a previously described materno-fetal PBPK model composed of integrated whole-body maternal model and multi-compartmental fetal model (Supplementary Figure 1) (28). The model was implemented in Simbiology® (v. 9.5, MATLAB® 2018b, Mathworks Inc., Natick, Massachusetts, USA) and extended to include the lymphatic circulation (Figure 1). Organ weights in the maternal model were predicted anthropometrically using the population physiology model described by Bosgra et al. (31). The compartments represented in the fetal model included the placenta, the amniotic fluid, fetal kidney, fetal liver, and fetal brain. Other organs were lumped together and represented by a single compartment as previously described (32). The structure of the fetal circulatory system was based on a published description (33) and organ blood flows were modeled using equations described by Zhang et al. (32).

Efavirenz, dolutegravir, and rilpivirine were selected for this study because they are approved for use in pregnancy and there is sufficient clinical data available on the pharmacokinetics of these drugs in pregnancy. Values of parameters representing the physicochemical properties of the study drugs (efavirenz, dolutegravir, and rilpivirine) such as octanol-water partition coefficient, acid dissociation constant and blood-to-plasma ratio, as well as their intrinsic hepatic clearances were obtained from literature (Table 1). Maternal and fetal anatomical and physiological adaptations to pregnancy were accounted for by the use of gestational-age dependent parameters where relevant (41). In some cases where necessary parameter values were not reported, published graph-plots of changes in the parameters: the placental thickness (18), rates of blood flow through the foramen ovale and ductus arteriosus (42), over the course of pregnancy were digitized (Plotdigitizer® version 2.6.6, Free Software Foundation, Boston, MA, USA). The data points obtained were used to generate equations of best-fit which were subsequently inputted into the model as previously described (28). Sensitivity analyses was conducted to observe the extent in which uncertainty in placental diffusion constant propagated into the fetal plasma predictions in the model (Supplementary Figure 2).

**Table 1:** Drug-specific parameters for efavirenz, dolutegravir, and rilpivirine used in building the lymph-PBPK model.

**Parameters**	**Description**	**Efavirenz (34)**	**Dolutegravir (34)**	**Rilpivirine (34)**
MW (g)	Molecular weight	316	419	366
Log P_ow_	Octanol-water partition coefficient	4.60	2.20	4.32
pKa	Dissociation constant	10.2	8.3	3.26
R	Blood-to-Plasma ratio	0.74	0.535	0.67
PSA (Å^2^)	Polar Surface Area	38.33	–	–
HBD	Hydrogen Bond Donor	1	–	–
f_u_	Fraction unbound	0.015	0.007	0.003
V_d_ (L/kg)	Volume of distribution	3.6	–	–
P_eff_ (10^−6^ cm/s)	Effective permeability (Caco-2)	2.5	–	12.0
ClintCYP2A6 (μL/min/pmol)	CYP2A6 Intrinsic Hepatic clearance	0.08	–	–
ClintCYP2B6 (μL/min/pmol)	CYP2B6 Intrinsic Hepatic clearance	0.55	–	–
ClintCYP1A2 (μL/min/pmol)	CYP1A2 Intrinsic Hepatic clearance	0.07	–	–
ClintCYP3A4 (μL/min/pmol)	CYP3A4 Intrinsic Hepatic clearance	0.007	3.0	2.04
ClintCYP3A5 (μL/min/pmol)	CYP3A5 Intrinsic Hepatic clearance	0.03	–	–
ClintUGT1A1 (μL/min/pmol)	UGT1A1 Intrinsic Hepatic clearance	–	3.2	–
d	Particle size (Mean ± SD)	2.35 ± 0.48 μm (35)	5.7 μm (36)	200 nm (37)
Plasma MEC (μg/mL)	Minimum Effective Concentration	8E-1 (38)	3.0E-1 (39)	5.0E-2 (40)
*In-vitro* adjusted PBIC (μg/mL)	Protein Binding Inhibitory Concentration	1.26E-1	6.40E-2	2.03E-2
Water Solubility (mg/mL)	Water solubility at 25°C	0.093	0.095	0.094

### Modeling the Lymphatic Circulation

The lymph node draining each organ was collected into a central lymph node compartment. The lymph returns back to the venous circulation at 1.7% rate of cardiac output to maintain body fluid balance (33, 43). The lymph flow and volume of extracellular water for each organ represented in the model is presented in Table 2 (44). Small drug molecules disintegrating from formulation matrix were assumed to be equilibrated between plasma and interstitial fluid (45). The model assumed transfer of drug from interstitial fluid into the lymphatic circulation by diffusion due to low transporter expression in the lymph nodes (16). The diffusion process was described by adapting Fick's diffusion equation (46) as shown below:

(1) $$\begin{array}{r} Q_{lymph,\;drug}=\frac{k_{drug}\times TSA_{lymph}\times f_{u}\times \left(C_{int}-C_{lym}\right)}{LT} \end{array}$$

where *k*_*drug*_ is the diffusion coefficient of the drug, *TSA*_*lymph*_ is the total surface area of initial lymphatics, *f*_*u*_ is the fraction of unbound drug, (*C*_*int*_ − *C*_*lym*_) is the drug concentration gradient across interstitial-lymph barrier, and *LT* is the wall thickness of initial lymphatics.

**Table 2:** Lymph flow draining various tissues in the human body (44).

**Tissues**	**Lymph flow (% CO)**	**Fraction of extracellular water**
Adipose	12.8	0.141
Bone	0.00	0.098
Brain	1.05	0.092
Gut	12.0	0.267
Heart	1.00	0.313
Kidney	8.50	0.283
Liver	33.0	0.165
Lung	3.00	0.348
Muscle	16.0	0.091
Pancreas	0.30	0.120
Skin	7.30	0.623
Spleen	0.00	0.208
Subcutaneous	0.04	0.623

*CO, Cardiac output*.

The diffusion coefficient of each drug, *k*_*drug*_, was calculated based on the Stokes-Einstein equation (47), as shown below:

(2) $$\begin{array}{r} k_{drug}=\frac{RT}{6\pi \times N_{a}\times r_{drug}\times \eta } \end{array}$$

where *RT* is the product of gas constant and body temperature at 37°C = 2.5 × 10^5^ Ncm/mol, *N*_*a*_ is the Avogadro's constant = 6.022 × 10^23^ /mol, *r*_*drug*_ is radius of drug, and η is the viscosity of water = 1.17 × 10^−9^ Nmin/cm^2^.

Lymph is collected by diffusion through the initial lymphatics in various organs. The shape of initial lymphatics was modeled to be a cone with a closed smaller end because it has a blinded (closed) end with a small diameter, which increases along the lymphatic vessels up to the pre-collecting lymphatic vessels (47, 48). The formula for the surface area of a cone was used to represent the surface area of a lymphatic vessels, *SA*_*lymph*_, as shown in the equation below:

(3) $$\begin{array}{r} SA_{lymph}=\pi r_{il}\left(r_{il}+\sqrt{{l_{il}}^{2}+{r_{il}}^{2}}\right) \end{array}$$

where *r*_*il*_ and *l*_*il*_ are the radius and length of the initial lymphatic vessel, respectively. The mean (±standard deviation) diameter and length of the closed end of an initial lymphatics had earlier been determined to be 30.8 ± 9.5 μm and 834 ± 796 μm, respectively (48). The suggested number of lymph nodes in the body is 500–600 (43), it was therefore assumed that the total surface area of initial lymphatic vessels, *TSA*_*lymph*_, is 500 times the surface area of an initial lymphatic vessel. The wall thickness of initial lymphatics has been reported to be in the range of 50–100 nm (47).

Absorption, distribution, metabolism and elimination were modeled as previously described for the base model (28). Previously undescribed model equations are presented in Supplementary Table 1 for reference.

### Model Verification and Model Simulation

Model predictions for key system parameters, including organ weights and blood flow, were compared with published reference values (41, 49–51). Published clinical pharmacokinetic studies on efavirenz, dolutegravir, and rilpivirine during pregnancy were searched through PubMed using combinations of drug name, pharmacokinetics, fetal exposure, infant washout, and pregnancy as keywords. In each case, the predicted steady-state pharmacokinetic parameters computed from simulated concentration-time data were compared with published data. Importantly, to ensure that the introduction of the lymphatic model into our previously published materno-fetal model does not affect key predictions, the model was revalidated for key system parameters relevant to drug disposition, and pharmacokinetic parameters in virtual populations of non-pregnant adults and pregnant women. To facilitate validation against clinical pharmacokinetic data from non-pregnant adult populations, non-pregnant equivalent of relevant model parameters and equations describing key processes were created. This allowed for easy activation/deactivation of model equations for the pregnant population while running simulation for the non-pregnant population. An absolute average fold error of <2.0 in predictions when compared with clinical data was set as acceptance threshold for model verification.

Verified models were used to predict the lymph and fetal concentration-time profiles of efavirenz, dolutegravir and rilpivirine following 100% adherence to therapy. Each simulation consisted of a virtual population of 50 females, non-pregnant or pregnant. Study drugs were administered orally at standard doses, 600 mg for efavirenz, 50 mg for dolutegravir, and 25 mg for rilpivirine. Concentration-time data were collected at steady state over a 24 h dosing period at 30 min and then hourly. Infant washout delivery was modeled by dose cessation in the maternal PBPK submodel. The extent of exposure to study drug was calculated as the ratio of AUC in compartments of interest within the same time interval. Non-adherence was modeled by dose cessation at steady state. Dose forgiveness was estimated in lymph and fetal compartment as the time it takes for drug concentration to decrease below the published minimum effective concentration (MEC) after the last dosing interval for each drug: 800 for efavirenz, 300 for rilpivirine and 50 ng/ml for dolutegravir (52–54).

Essential pharmacokinetic parameters, including Cmax, Cmin, and AUC (0–24) at steady state, for both maternal lymph and fetal compartments were computed from the corresponding concentration-time data. Dose input was stopped at delivery, and infant plasma exposure was predicted by measuring drug concentration 2–10 h post dose.

## Result

### Model Validation

**Figure 2:**
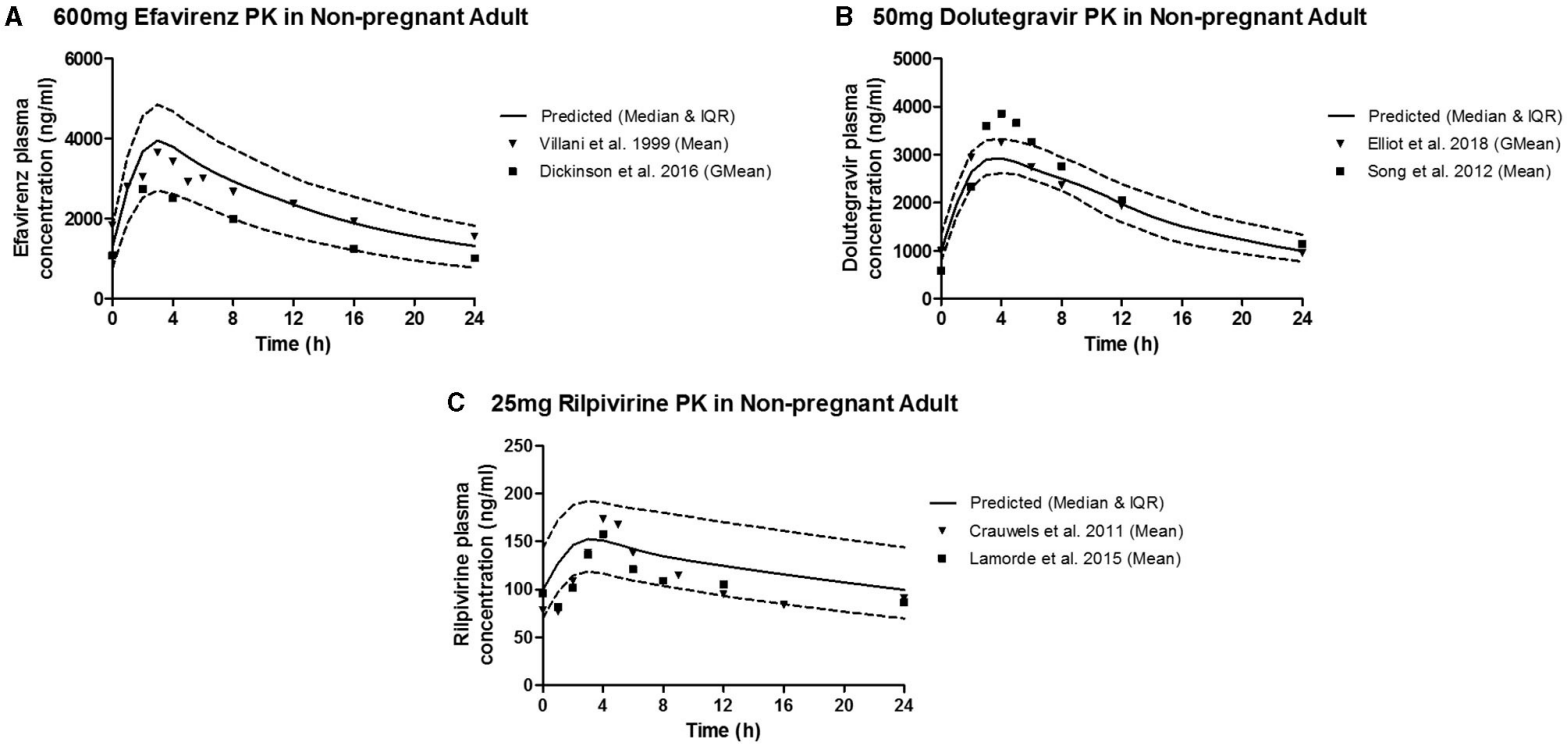
Median (IQR) Predicted vs. Observed plasma concentration-time profile following standard dose of **(A)** 600 mg efavirenz, **(B)** 50 mg dolutegravir, and **(C)** 25 mg rilpivirine in non-pregnant adults.

**Figure 3:**
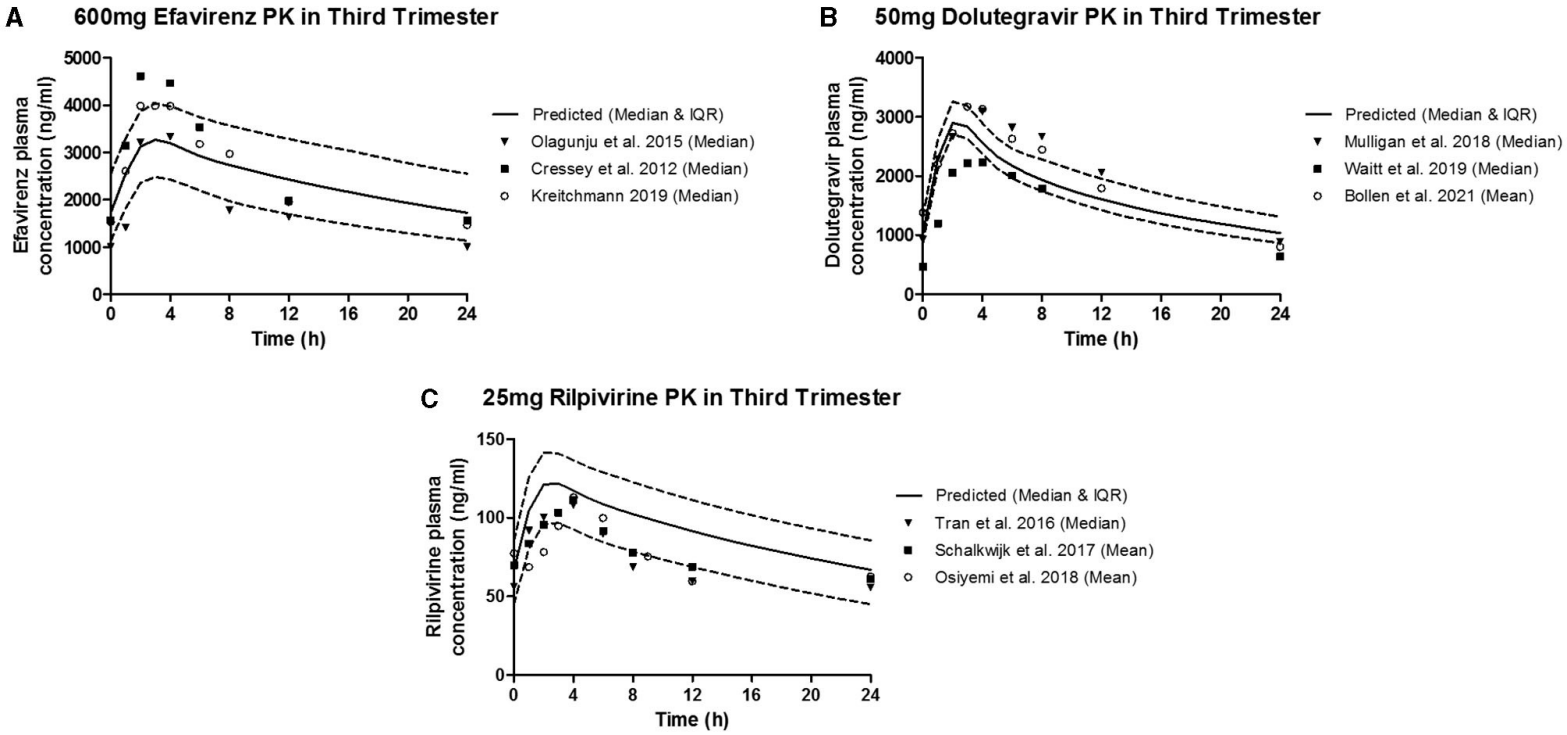
Median (IQR) Predicted vs. Observed plasma concentration-time profile following standard dose of **(A)** 600 mg efavirenz, **(B)** 50 mg dolutegravir, and **(C)** 25 mg rilpivirine in third trimester.

The predicted plasma pharmacokinetic parameters in the non-pregnant adult model were within 1.19–1.80 average fold difference of clinically observed data (Table 3). Predicted plasma concentration-time curves were superimposed on clinically observed plasma concentration-time profiles (Figure 2) of each drug to visually assess predictive performance of the model. There is lack of clinical data to validate the lymph pharmacokinetics predictions. Maternal plasma pharmacokinetic predictions of the m-f-PBPK model developed were validated with clinically observed pharmacokinetics data in third trimester, and were within 1.13–1.76 average fold difference of clinically observed data (Table 4). The predicted concentration-time profiles were comparable with clinically observed maternal plasma concentration-time profiles in third trimester (Figure 3).

**Table 3:** Median Plasma and Lymph pharmacokinetic parameters for efavirenz, dolutegravir, and rilpivirine in non-pregnant adult.

	**Plasma**			**Lymph**		**Lymph-to-plasma ratio**	
	**Predicted**	**Observed**	**AAFE**	**Predicted**	**Observed**	**Predicted**	Reported^*^
**Efavirenz 600 mg**							
AUC_css, 0−24/∞_ (nghr/mL)	57,763	56,630 (55), 67,200 (56)	1.46, 1.48	408,184	–	7.07	0.86–7.14 (16, 24)
C_max, css_ (ng/mL)	3,950	3,659 (55), 3,660 (56)	1.36, 1.36	22,497	–		
C_24, css_ (ng/mL)	1,315	1,557 (55), 1,820 (56)	1.70, 1.80	11,285	–		
**Dolutegravir 50 mg**							
AUC_css, 0−24/∞_ (nghr/mL)	46,114	47,137 (57), 50,300 (58)	1.24, 1.26	765.7	−	0.017	0.082 (24)
C_max, css_ (ng/mL)	2,924	3,250 (57), 2,650 (58)	1.18, 1.19	46.3	−		
C_24, css_ (ng/mL)	992.0	950.0 (57), 750.0 (58)	1.46, 1.55	17.4	−		
**Rilpivirine 25 mg**							
AUC_css, 0−24/∞_ (nghr/mL)	2,981	2,526 (59), 2,582 (60)	1.46, 1.45	4,653	−	1.57	>1 (24, 37)
C_max, css_ (ng/mL)	152	173 (59) 175 (60)	1.36, 1.37	225	–		
C_24, css_ (ng/mL)	97.2	91.0 (59), 92.0 (60)	1.57, 1.57	161	–		

*AAFE, Absolute Average Fold Error*.

* *Lymph-to-plasma ratios in vitro, ex vivo, and animal studies*.

**Table 4:** Pharmacokinetic parameters of efavirenz, dolutegravir, and rilpivirine in third trimester of pregnancy and infant washout after delivery.

	**Maternal plasma (3rd trimester)**				**Infant washout after delivery**		
	**Predicted**	**Observed**	**AAFE**		**Predicted**	**Observed**	**AAFE**
**Efavirenz 600 mg**							
AUC_css, 0−24/∞_ (ng.h/mL)	58,120	42,943 (61), 55,400 (62), 60,020 (63)	1.57, 1.47, 1.46	C_2−10h_	1,016	1,100 (63)	1.08
C_max, css_ (ng/mL)	3,270	3,331 (61), 5,440 (62), 5,130 (63)	1.33, 1.76, 1.68				
C_24, css_ (ng/mL)	1,724	1,002 (61), 1,600 (62), 1,480 (63)	1.99, 1.66, 1.68				
**Dolutegravir 50 mg**							
AUC_css, 0−24/∞_ (ng.h/mL)	41,166	40,800 (64)^§^, 49,119 (2), 35,322 (65)	1.18, 1.24, 1.24	C_2−10h_	907.4	1,730 (2)	1.91
C_max, css_ (ng/mL)	2,899	3,150 (64), 3,137 (2), 2,534 (65)	1.13, 1.13, 1.18				
C_24, css_ (ng/mL)	1,035	1,000 (64), 921.5 (2), 642 (65)	1.28, 1.30, 1.68				
**Rilpivirine 25 mg**							
AUC_css, 0−24/∞_ (nghr/mL)	2,205	1,684 (65), 1,762 (3)^§^, 1,710 (66)^§^	1.46, 1.43, 1.45	C_2−10h_	44.59	–	–
C_max, css_ (ng/mL)	121.8	108 (65), 123 (3), 110 (66)	1.29, 1.26, 1.28				
C_24, css_ (ng/mL)	66.9	56 (65), 53 (3), 50 (66)	1.55, 1.58, 1.61				

*AAFE, Absolute Average Fold Error. All values are reported in median;*

§ *Mean values*.

Obtaining fetal plasma pharmacokinetic parameters for many drugs during clinical studies remains a challenge, but some clinical data on concentration of efavirenz and dolutegravir in non-breastfed infants shortly after delivery (2–10 h) are available (2, 63). However, the time of delivery was not reported in any of these studies. Thus, a delivery time of 12:00 after the last maternal dose was assumed in the model. The model-predicted infant plasma concentration 2–10 h post-delivery was similar to the reported infant concentration for efavirenz and dolutegravir within the same period. The model-predicted infant median concentration for rilpivirine 2–10 h post-delivery was 44.59 ng/mL.

The result of the sensitivity analysis showed that placental diffusion constant is a significant parameter that affect movement of drugs studied into the fetal compartment (Supplementary Figure 2). Also, possible influence of the lymph component on accuracy of prediction in the full m-f-PBPK model was evaluated. The median maternal and fetal plasma AUC (0–24) for efavirenz (58,120 vs. 51,984 ng.h/ml; and 34,404 vs. 30,864 ng.h/ml) in the full m-f-PBPK model was similar to predictions in the m-f-PBPK model without lymphatic component (*data not shown*).

### Model Predictions

**Figure 4:**
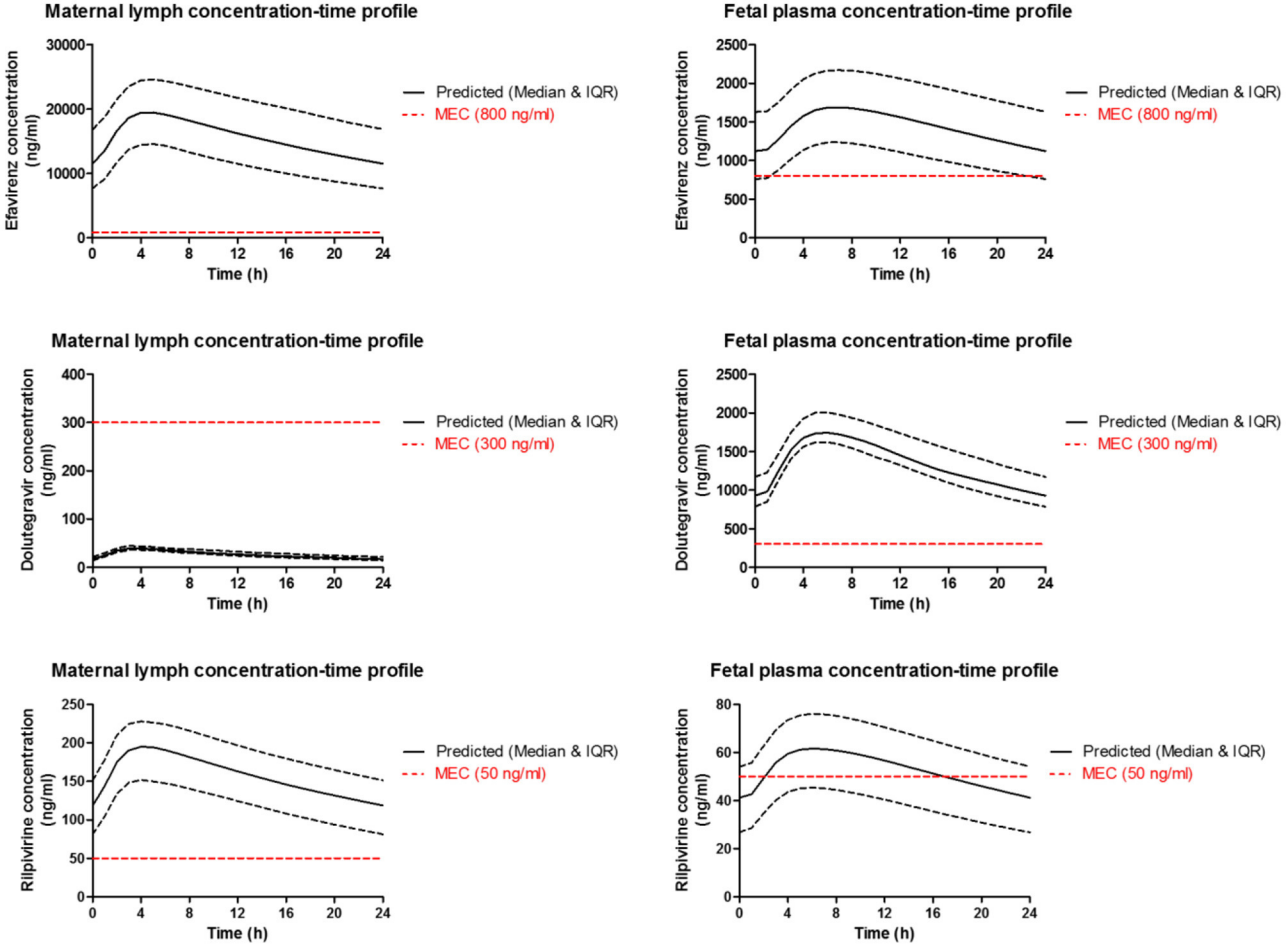
Median (IQR) Predicted maternal plasma and lymph; and fetal plasma concentration-time profile following standard dose of 600 mg efavirenz, 50 mg dolutegravir, and 25 mg rilpivirine in third trimester. Dotted line represent the reported minimum effective concentration (MEC) for each drug at the standard dose.

Simulation was run for 50 virtual patients with mean ± SD age and gestational age of 29 ± 12 years and 39 ± 2.25 weeks, respectively. Each virtual patient was administered a single dose of 600 mg of efavirenz, 50 mg of dolutegravir, or 25 mg of rilpivirine. Concentration-time data was collected after reaching steady state. The validated model was employed to predict the maternal lymph and fetal plasma pharmacokinetics of 600 mg, 50 mg and 25 mg daily dose of efavirenz, dolutegravir, and rilpivirine, respectively (Figure 4). The maternal lymph-to-plasma and fetal-to-maternal plasma AUC ratios of 0.592, 0.781, and 0.573 were obtained for efavirenz, dolutegravir and rilpivirine, respectively (Table 5). Efavirenz was predicted to accumulate in maternal lymph by over 6-folds and rilpivirine accumulated by <2-folds. Poor lymph penetration was predicted for dolutegravir with only 1.6% of plasma dolutegravir entering the lymph. Predictions of fetal plasma concentrations of efavirenz, dolutegravir, and rilpivirine were 59.2, 78.1, and 57.3% of maternal plasma concentrations, respectively.

**Table 5:** Predicted median (IQR) maternal plasma and lymph in third trimester; and fetal plasma pharmacokinetic parameters of efavirenz, dolutegravir and rilpivirine.

	**Pharmacokinetic parameters**		
	**AUC (ng.h/mL)**	**Cmax (ng/mL)**	**C24 (ng/mL)**
**Efavirenz 600 mg (** ***n*** **= 50)**			
Maternal Plasma	58,120 (41,149–78,030)	3,270 (2,478–4,035)	1,724 (1,132–2,547)
Maternal Lymph	373,790 (264,477–502,688)	19,470 (14,560–24,579)	11,514 (7,666–16,852)
Lymph-to-plasma AUCratio	6.431		
Fetal Plasma	34,404 (24,236–46,364)	1,689 (1,235–2,170)	1,123 (758.4–1,630)
Fetal-to-plasma ratio	0.5919		
**Dolutegravir 50 mg (** ***n*** **= 50)**			
Maternal Plasma	41,166 (36,660–48,827)	2,899 (2,707–3,259)	1,035 (865.3–1,313)
Maternal Lymph	643.7 (571.7–759.9)	39.26 (35.74–44.01)	16.96 (14.29–21.49)
Lymph-to-plasma ratio	0.0156		
Fetal Plasma	32,152 (28,905–38,541)	1,742 (1,620–2,007)	927.4 (784.7–1,170)
Fetal-to-plasma ratio	0.781		
**Rilpivirine 25 mg (** ***n*** **= 50)**			
Maternal Plasma	22,05 (1,649–2,674)	121.8 (96.71–141.4)	66.91 (45.01–85.67)
Maternal Lymph	3,788 (2,841–4,592)	195.1 (151.8–227.8)	118.8 (81.43–151.4)
Lymph-to-plasma ratio	1.717		
Fetal Plasma	1,263 (888.5–1,591)	61.66 (45.46–76.0)	41.26 (26.83–54.11)
Fetal-to-plasma ratio	0.573		

Dose forgiveness of 600 mg efavirenz, 50 mg dolutegravir, and 25 mg rilpivirine were determined in order to estimate the time it takes for the drug concentration to persist above the MEC in the maternal lymph and fetal plasma after dose cessation in third trimester. Model-predicted efavirenz and rilpivirine maternal lymph concentration remained above MEC for 150 and 56 h, respectively, but dolutegravir concentration was persistently below the MEC. In fetal plasma however, dolutegravir and efavirenz were above MEC for 36 and 66 h, respectively, but rilpivirine concentration persisted below the MEC (Table 6).

**Table 6:** Predicted maternal lymph and fetal plasma dose forgiveness of 600 mg efavirenz, 50 mg dolutegravir and 25 mg rilpivirine during third trimester.

***n* = 50**	**Efavirenz**	**Dolutegravir**	**Rilpivirine**
**Maternal Lymph**			
Duration of action (h)	150	0	56
Dosing interval (h)	24	24	24
Forgiveness (h)	126	0	32
Forgiveness index	5.25	0	1.33
**Fetal Plasma**			
Duration of action (h)	36	66	0
Dosing interval (h)	24	24	24
Forgiveness (h)	12	42	0
Forgiveness index	0.5	1.75	0

## Discussion

Our extended m-f-PBPK model which incorporated lymphatic circulation into an existing whole-body pregnancy model was successfully used to predict maternal lymph and fetal plasma pharmacokinetics of the ARVs efavirenz, dolutegravir, and rilpivirine. Model predictions for maternal plasma pharmacokinetics during the third trimester and infant delivery drug exposures were within 1.08–1.99 average fold difference of clinical data. Predicted maternal lymph-to-plasma AUC ratio was highest for efavirenz at 6.4, followed by rilpivirine at 1.7 and lowest for dolutegravir at 0.016. Model-predicted fetal plasma-to-maternal plasma AUC ratios were 0.59 for efavirenz, 0.78 for dolutegravir, and 0.57 for rilpivirine. The median predicted lymph concentration at 24 h after dose was above the published MEC for efavirenz and rilpivirine only. The predicted low lymphoid tissue penetration of dolutegravir appears to be significantly counterbalanced by its extended dose forgiveness (42 h compared with 12 h for efavirenz and 0 h for rilpivirine) and adequate fetal compartment exposure. Hence, it is unlikely to be a predictor of maternal virological failure or mother-to-child transmission risks.

Although ART suppresses plasma viraemia below the limit of detection, persistence of latent but replication-competent HIV in sanctuary tissues during active treatment constitutes a challenge in HIV cure research (38). Despite lymphoid tissues having the highest proportion of latent HIV (14, 15), no comprehensive assessment of lymph pharmacokinetics of ARVs in humans (pregnant and non-pregnant) is available due to the invasiveness of the conventional lymph node aspiration technique. Although, a number of studies have used *in-vitro, ex-vivo*, and *in vivo* animals models to determine lymphatic exposure of efavirenz, dolutegravir, and rilpivirine (16, 24, 67), such models are known to be inadequate representations of what is expected in humans. It is known that suboptimal adherence to ART may lead to subtherapeutic drug levels in the systemic circulation, stimulating latent HIV in lymphoid tissues to resume active replication, thereby causing viral rebound (19). Detectable viral load is a known risk factor for MTCT and optimal adherence during pregnancy remains critical.

Administration of ARVs during pregnancy has several benefits, notably PMTCT which may be partly due to fetal prophylactic pre-exposure to ARVs. Past studies have relied on umbilical cord blood concentration at delivery to measure the extent of fetal exposure of ARVs. This method has limitations such as single time-point measurement and sample-time variation relative to maternal dosing. In this study, the infant plasma concentration prediction was validated with efavirenz and dolutegravir clinical data for infant washout in non-breastfed babies 2–10 h post-delivery. Post-delivery scenarios were simulated by stopping maternal dosing at term, and then estimating median fetal concentration after 2–10 h. The results were within the acceptable 2-fold difference for efavirenz and dolutegravir. The validated model was applied to rilpivirine and its fetal plasma concentration-time profile was also predicted successfully. The predicted fetal-to-maternal plasma ratio of efavirenz, dolutegravir, and rilpivirine were 0.591, 0.781 and 0.573, respectively. The predicted median fetal concentration at 24 h was higher than MEC for efavirenz (1,123 vs. 800 ng/mL) and dolutegravir (927.4 vs. 300 ng/mL), but lower for rilpivirine (41.26 vs. 50 ng/mL). Differential concentrations of efavirenz, dolutegravir and rilpivirine in maternal lymph and fetal plasma is, to a certain extent, as a result of differences in their physicochemical properties such as plasma protein binding, log P, molecular weight, and pKa (68–70). For instance, high pKa, log P and hydrophobicity of efavirenz were identified to be responsible for high penetration of efavirenz into human lymphoid endothelial cells compared to dolutegravir (24).

Our present study predicted C_trough_ of 992 ng/mL and AUC of 46,114 ng/mL for dolutegravir in non-pregnant women, these are similar to predictions by Freriksen et al. (71), and Liu et al. (72) respectively, and are within 1.5-fold error to clinically observed data of the drug. This further indicated a strong reliability in the non-pregnant model and the confidence to extend it to incorporate the pregnancy model. Furthermore, the maternal dolutegravir PK parameters during pregnancy predicted by the model employed in this present study were similar to those predicted by Liu et al. (72). Additionally, the predicted fetal exposure to dolutegravir in the present study was comparable to that reported by Freriksen et al. (71). Although, fetal-to-maternal AUC plasma exposure ratio was predicted in our study, it was assumed to be similar to and within the range of the cord blood-to-maternal blood concentration ratios predicted in their study and observed clinical data. Likewise, the C_max_ and the C_min_ of the maternal plasma concentration predicted by our model was lower and higher, respectively, in comparison to their reported values (71). Further studies are still suggested to establish this assumption of similarity.

Dose forgiveness was used to estimate how long it would take for drug concentration in maternal lymph and fetal plasma to reduce below MEC in non-adhering pregnant mothers. Efavirenz and rilpivirine lymph concentrations remained above MEC for 126 and 32 h, respectively; efavirenz may therefore offer a longer protection in lymph against latent HIV in non-adhering pregnant women. While this may be an advantage, the ability of wild-type HIV to develop resistance to efavirenz monotherapy is of concern (67). Therefore, further investigation is required to know the extent of lymph exposure of tenofovir and emtricitabine which are commonly used in combination with efavirenz. In fetal plasma, efavirenz and dolutegravir concentration remained above MEC for 12 and 42 h, respectively. These results showed that efavirenz and rilpivirine may offer adequate protection against viral rebound from the maternal lymph nodes, but in rare situations where the virus enters the systemic circulation, efavirenz and dolutegravir may offer adequate fetal pre-exposure prophylaxis. Longer dose forgiveness of dolutegravir in fetal plasma offers sustained pre-exposure prophylaxis to fetus in pregnant women with suboptimal adherence. These results do not reflect the enzyme induction or inhibition effect of other drugs used as combination therapy. Therefore, interpretation of these results may be limited clinically.

Although, the current model reliably predicted lymphatic and fetal exposure to efavirenz, dolutegravir, and rilpivirine during the third trimester, a number of limitations are identifiable. Firstly, our model did not account for the possible role of transporter activities in placental drug transfer due to lack of sufficient data for model parameterization. The use of data from cell lines expressing relevant transporters such the BeWo monolayer are possible options to mechanistically describe these processes. Unfortunately, these data are not currently available in the literature and thus the option of relying only on passive processes for our predictions. Additionally, such models do not adequately recapitulate these processes in humans. The inclusion of drug transporters and associated variability in their expression can potentially improve the accuracy of model predictions, particularly for drugs that are substrates for these transporters. Secondly, due to lack of data we relied on key assumptions supported by sensitivity analyses for placental diffusion constants of study drugs. Model predictions were fitted to clinically observed infant plasma concentration at delivery. While this resulted in adequate predictions of infant exposure of the study drugs at delivery, further enhancement is desirable in future studies. Thirdly, there was no previous study to validate rilpivirine infant washout data. The validated model with available clinical data on infant washout for efavirenz and dolutegravir was extended to predict for rilpivirine. Furthermore, there are no clinical data available in humans to validate the predictions of the lymphatic model. Although lymph exposure data are available from *ex-vivo, in-vitro*, and animal studies, we could not rely on them to assess the predictive performance of the lymphatic model due to well-established inter-species variation.

In conclusion, predictions from our extended m-f-PBPK model showed differences in the distribution of efavirenz, rilpivirine, and dolutegravir into the lymph during pregnancy and the fetal compartment. Importantly, the inclusion of dose forgiveness predictions indicate alignment with recommendations of no dose adjustment despite moderate changes in exposure during pregnancy observed in clinical studies. This is an important new application of PBPK modeling strategy to evaluate the adequacy of drug exposure in an otherwise inaccessible compartment.

## Data Availability Statement

The original contributions presented in the study are included in the article/Supplementary Material, further inquiries can be directed to the corresponding author/s.

## Author Contributions

All co-authors contributed equally to the conception of the ideas presented here, the conduct of the research, and the preparation of this manuscript.

## Conflict of Interest

The authors declare that the research was conducted in the absence of any commercial or financial relationships that could be construed as a potential conflict of interest.

## Publisher's Note

All claims expressed in this article are solely those of the authors and do not necessarily represent those of their affiliated organizations, or those of the publisher, the editors and the reviewers. Any product that may be evaluated in this article, or claim that may be made by its manufacturer, is not guaranteed or endorsed by the publisher.
