# Supplementary material for: Mechanistic Modeling of Maternal Lymphoid and Fetal Plasma Antiretroviral Exposure During the Third Trimester
Source: Front Pediatr. 2021 Sep 20;9:734122. doi: 10.3389/fped.2021.734122 (PMC8488224; doi:10.3389/fped.2021.734122)
Supplement: Supplementary file 1 [file Data_Sheet_1.PDF]

List of equations

S/N	Equations
1	$P_{eff} = 10^{(0.6836\ x\ (log\ Caco-2)) - 0.5579)}$
2	$P_{eff} = 10^{(-2.546 - 0.011\ (PSA) - 0.278\ (HBD))}$
3	$K_a = \frac{2\ P_{eff}}{R}$
4	$Pt{:}p, nonadipose = \frac{[Po{:}w\ x\ (Vnlt+\ 0.3\ x\ Vpht)]+[1\ x\ (Vwt+0.7\ x\ Vpht)]}{[Po{:}w\ x\ (Vnlp+\ 0.3\ x\ Vphp)]+[1\ x\ (Vwp+0.7\ x\ Vphp)]} \times \frac{fu,p}{fu,t}$
5	$Pt{:}p, adipose = \frac{[Dvo{:}w\ x\ (Vnlt+\ 0.3\ x\ Vpht)]+[1\ x\ (Vwt+0.7\ x\ Vpht)]}{[Dvo{:}w\ x\ (Vnlp+\ 0.3\ x\ Vphp)]+[1\ x\ (Vwp+0.7\ x\ Vphp)]} \times \frac{fu,p}{1}$
6	$V_{ss} = (\Sigma Vt * Pt{:}p) + (Ve * E{:}P) + Vp$
7	$F_{u,p} = \frac{1}{1+(K_{p,pp} \times [P_{pp}]\ )}$
8	$K_{p,pp} = \frac{(1-f_u\ )}{(69.7 \times f_u)}$
9	$CL = \frac{V_d \times 0.693}{t_{1/2}}$
10	$Q_{pl,drug} = \frac{K \times SA_{pv} \times f_u \times (C_1 - C_2)}{PT}$
11	$K_{THAL} = \frac{P_{eff,THAL} \times K_{EFV}}{P_{eff,EFV}}$
12	Qpv (L/hr) = 0.714 + 0.0489 GA + 0.0008 GA^2
13	Qda (L/hr) = 0.0056 GA + 0.1441
