# Supplementary material for: Mechanistic Modeling of Maternal Lymphoid and Fetal Plasma Antiretroviral Exposure During the Third Trimester
Source: Front Pediatr. 2021 Sep 20;9:734122. doi: 10.3389/fped.2021.734122 (PMC8488224; doi:10.3389/fped.2021.734122)
Supplement: Supplementary file 2 [file Data_Sheet_2.PDF]

A.

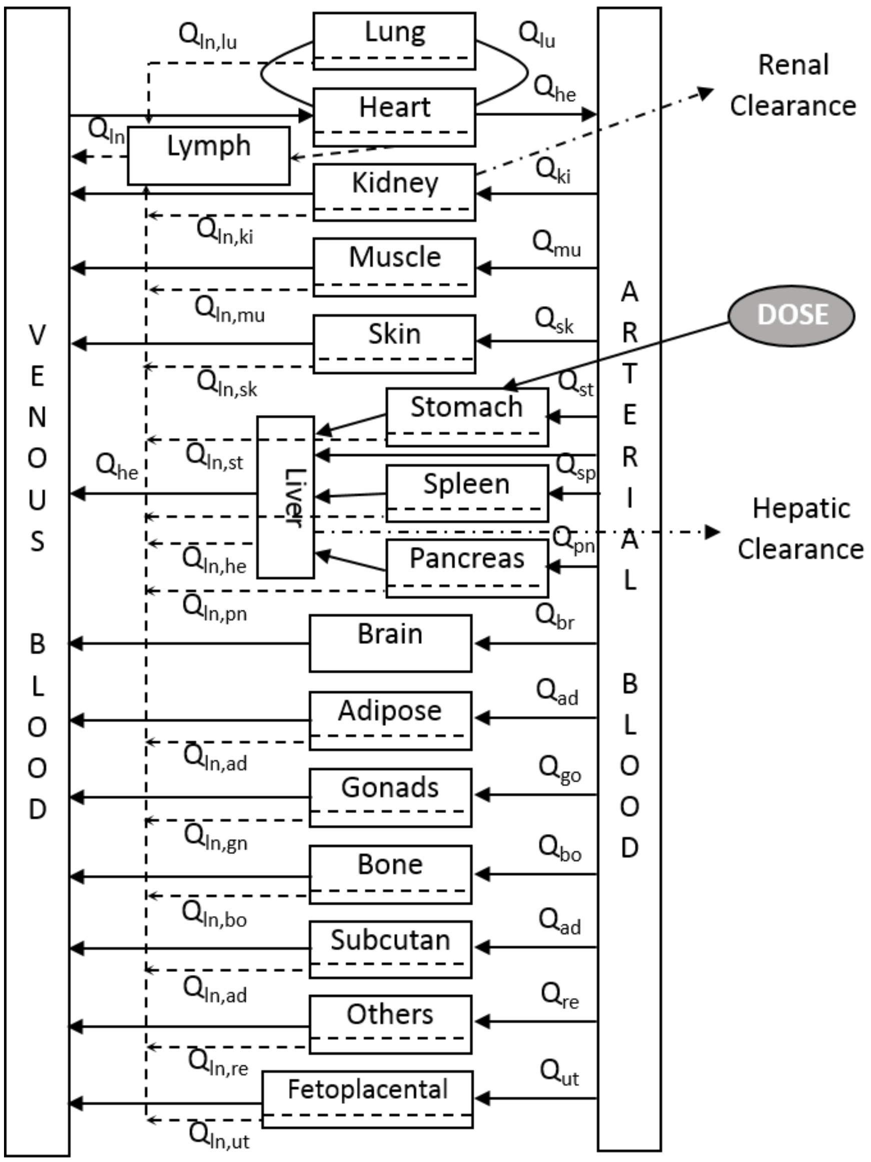

B.

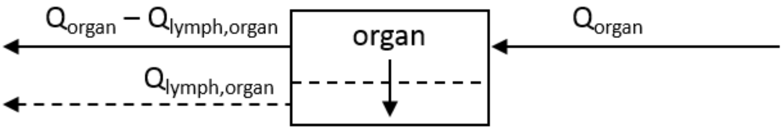

Figure S1: Schematic representation of (A) pregnancy PBPK model with lymphatic circulation, and (B) flow of drugs into lymph from tissues/organs in the model. The fetoplacental compartment presented a simplified representation of fetal compartment in the actual model.

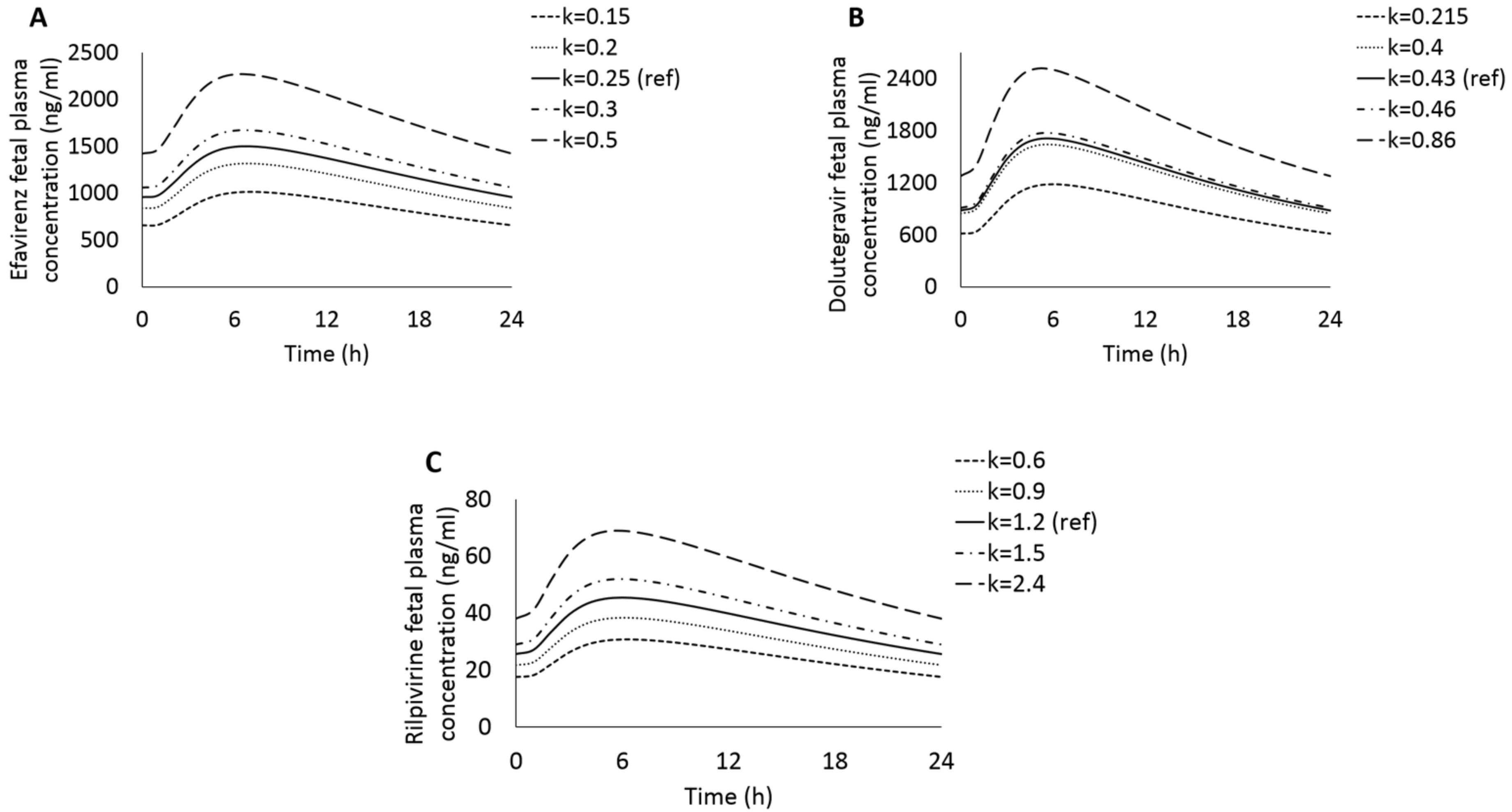

Figure S2: Sensitivity analysis of (A) efavirenz, (B) dolutegravir and (C) rilpivirine diffusion constant used in the model to fetal plasma predictions.

Table S1: Equations describing absorption, distribution and elimination

Physiological Process	Parameter	Equation
Absorption	Effective Permeability	$P_{eff} = 10^{(0.6836\times(\log Caco-2)-0.5579)}$
		$P_{eff} = 10^{\left(-2.546-0.011(PSA)-0.278(HBD)\right)}$
	Absorption rate constant	$K_a = \frac{2\,P_{eff}}{R}$
Distribution	Partition coefficient	$Pt{:}p, nonadipose = \frac{[Po{:}w \times (Vnlt + 0.3 \times Vpht)] + [1 \times (Vwt + 0.7 \times Vpht)]}{[Po{:}w \times (Vnlp + 0.3 \times Vphp)] + [1 \times (Vwp + 0.7 \times Vphp)]} \times \frac{f_{u,p}}{f_{u,t}}$
		$Pt{:}p, adipose = \frac{[Dvo{:}w \times (Vnlt + 0.3 \times Vpht)] + [1 \times (Vwt + 0.7 \times Vpht)]}{[Dvo{:}w \times (Vnlp + 0.3 \times Vphp)] + [1 \times (Vwp + 0.7 \times Vphp)]} \times \frac{f_{u,p}}{1}$
	Volume of distribution	$V_{ss} = (\Sigma Vt * Pt{:}p) + (Ve * E{:}P) + Vp$
	Fraction unbound	$f_{u,p} = \frac{1}{1 + \left(K_{p,pp} \times [P_{pp}]\right)}$
		$K_{p,pp} = \frac{(1 - f_u)}{(69.7 \times f_u)}$
Metabolism and Elimination	Clearance	$CL = \frac{V_d \times 0.693}{t_{1/2}}$

PSA is Polar surface area; HBD is hydrogen bond donor; R is radius of small intestine; $Pt{:}p$ is plasma tissue:plasma partition coefficient; $Po{:}w$ is n-octanol:buffer partition coefficient of the non-ionised species at pH 7.4; $Dvo{:}w$ is olive oil:buffer partition coefficient of ionized and non-ionised species at pH 7.4; V is fractional tissue volume content of neutral lipids (nl), phospholipids (ph) and water(w); t is tissue and p is plasma; V_{ss} is Volume of distribution at steady state; V is fractional body volume of erythrocyte (e), plasma (p) and tissue (t); $E{:}P$ is erythrocyte:plasma ratio; f_u is the fraction of the unbound drug in non-pregnant adults; $[P_{pp}]$ is the concentration of plasma proteins in pregnancy; $f_{u,p}$ is the fraction of the unbound drug during pregnancy and $K_{p,pp}$ is the constant of association of the drug to plasma proteins; V_d is volume of distribution, $t_{1/2}$ is half-life.
